# Multiple primary head and neck cancers: a scenario-based framework for diagnosis and modern multidisciplinary management

**DOI:** 10.3389/fonc.2026.1841052

**Published:** 2026-06-05

**Authors:** Aleksandra Gerlach, Hanna Magdalena Ras, Wiktor Bonar, Krzysztof Składowski, Marek Zawadzki, Małgorzata Wierzbicka

**Affiliations:** 1 Wojewodzki Szpital Specjalistyczny we Wroclawiu, Wrocław, Poland; 2 Narodowy Instytut Onkologii im Marii Sklodowskiej-Curie Panstwowy Instytut Badawczy Oddzial w Gliwicach, Gliwice, Poland; 3 Politechnika Wroclawska, Wrocław, Poland

**Keywords:** head and neck squamous cell carcinoma (HNSCC), immune checkpoint inhibitors (nivolumab, pembrolizumab), intensity modulated radiotherapy (IMRT) and re-irradiation, multidisciplinary tumour board, multiple primary head and neck cancer, scenario-based management framework, synchronous and metachronous tumours

## Abstract

**Background:**

Multiple primary tumours (MPT) involving the head and neck represent a growing and insufficiently systematised challenge in contemporary head and neck oncology. Patients with head and neck squamous cell carcinoma (HNSCC) face a cumulative risk of 2–4% per year of developing a second primary malignancy, driven by field cancerisation, persistent exposure to tobacco and alcohol, and improved survival after multimodality treatment.

**Methods:**

Observational analysis of patients with ICD-10 C00–C14 and C30–C32 diagnoses treated in a university otolaryngology–head and neck surgery department over a two-year period (2024–2025), with specific focus on cases fulfilling criteria for MPT, was performed. Detailed clinical, pathological and treatment variables for the first and subsequent primaries were extracted and summarised, and illustrative cases were organised into a predefined 2 × 2 clinical matrix: (1) synchronous primaries confined to the head and neck; (2) synchronous HNSCC with an extra head and neck primary; (3) metachronous primaries within the head and neck; and (4) metachronous HNSCC with a non-head and neck malignancy.

**Results:**

Among 274 consecutive patients treated for HNSCC, 15 (5.5%) were identified as having MPT. These encompassed all four matrix scenarios and included combinations of laryngeal and oropharyngeal carcinomas, salivary gland tumours, hepatocellular carcinoma, prostate cancer and renal tumours. Across scenarios, optimal care required systematic diagnostic work-up (panendoscopy, high-quality cross-sectional imaging, meticulous histopathology), multidisciplinary discussion, and tailored use of contemporary modalities such as IMRT (including re-irradiation), transoral surgery and immune checkpoint inhibitors. In older, multi-morbid patients, comprehensive geriatric assessment emerged as pivotal for balancing curative intent against toxicity and functional outcome.

**Conclusions:**

Multiple primary head and neck cancers constitute a distinct, non-rare workload in tertiary practice and cannot be adequately addressed by isolated case reports or generic HNSCC pathways. A scenario-based framework, operationalised through a 2 × 2 clinical matrix with scenario-specific diagnostic and therapeutic checklists, provides a pragmatic tool to standardise evaluation, structure tumour board deliberations, and align increasingly complex IMRT and immunotherapy-era treatment options with patient prognosis, comorbidity, and values.

## Introduction

Multiple primary tumours (MPT) involving the head and neck are an increasingly common challenge in clinical practice. In patients with head and neck squamous cell carcinoma (HNSCC), the cumulative risk of developing a second primary malignancy (SPM) reaches 2-4% per year and is several-fold higher than in the general population (1–6). Field cancerisation, chronic exposure to tobacco and alcohol, and improved survival after modern multimodality treatment all contribute to this burden (7–11) (**Table 1**).

**Table 1:** Incidence of multiple primary tumors in head and neck cancer – selected studies.

Author, year, Population	Type of MPT		SPT depending on follow-up time	survival	Overall SPT rate:	Comments
Dhooge et al., (12), 127 patients with squamous cell carcinoma of the head and neck	Simultaneous	3.00%	–	–	13.50%	
	Synchronous	5,5 %	–			
	Metachronous	8.00%	–	–		
Ha et al., (13) 790 patients between 1993 and 2011	Synchronous	3,7 %		22.5% mortality (median follow up 25 months)	5,95 %	
	Metachronous	2,3 %				
Chuang et al., (14), 13 population-based cancer registries. 99,257 patients			<12months -1,24%		10,95 %	20-year cumulative risk of SPC after head and neck cancer was 36% ( 33% in mouth and tongue, 46% in hypopharyngeal)
			1-4years -4,01%			
			5-9years – 2,83%			
			>10y- 2,42%			
Diaz et al., (15) (SEER) database 1973–2008, 104,639 patients			Median SPT diagnosis 5,9 y		4,41 % (4.1% men and 5.3% women)	Patients who developed SPM <2 years were excluded, because these were considered more likely to recurrences
Batista et al., (16) 824 patients between January 1, 2015, and December 31, 2019.	Synchronous	n18	median follow-up was 25 months.	14 months	6,43%	five-year survival rate was 53% for patients with multiple HNSCCs and 47% for patients with at least one non-HNT
	Metachronous	n35		58 months		
Davies et al., (17) 662 patient with HPV-positive oropharyngeal cancer treated with definitive intensity-modulated RT from 1/2009 to 12/2020			RISM 19,4-140months 1,2% 5 years 0,37% 10 years 1,74% Disease recurrence 16.6%			RISM defined as tumor occurring >5 years from treatment; occurrence at a geographically distinct site or subsite; or a histopathologically distinct primary tumor (including HPV-negative status)
Stepan et al, (18) 412 patients with HPV-related OPSCC who underwent transoral resection +/- adjuvant therapy between 1996 and 2018	Synchronous	n3	59.5 months		4.90%	Risk of SPM was lower for patients receiving adjuvant radiation
	Metachronous	n17				

HNSCC, head and neck squamous cell carcinoma; H&N, head and neck; OPSCC, oropharyngeal squamous cell carcinoma; SPM, second primary malignancy; SPT, second primary tumor; UADT, upper aerodigestive tract; RT, radiotherapy; OS, overall survival.

Classical definitions by Warren and Gates and contemporary registry rules (IARC/IACR, SEER) help standardise the coding of multiple primaries but do not address the practical question of how to treat two or more distinct tumours in the same patient (19–21) (**Table 1**). At the same time, the therapeutic landscape of HNSCC has evolved dramatically. Intensity-modulated radiotherapy (IMRT) and image-guided techniques allow highly conformal dose distributions and better sparing of organs at risk, thereby improving functional outcomes compared with two-dimensional and three-dimensional techniques (22–28). Immune checkpoint inhibitors such as nivolumab and pembrolizumab have become standards of care in recurrent/metastatic HNSCC, based on CheckMate 141 and KEYNOTE-048, with durable survival benefits in subsets of patients (29, 30). IMRT-based re-irradiation has opened new salvage options for recurrent or second primary HNSCC in previously irradiated fields (22–28).

The aim of this study is to analyse cases of MPT within the overall cohort of patients with HNSCC treated in the Department of Otolaryngology-Head and Neck Surgery. Rather than focusing on individual case reports, this article proposes a scenario-based framework for multiple primary tumours (MPTs) in the context of head and neck cancer.

## Methodology

We analysed patients diagnosed with C00-C14 and C30-C32 who were treated in a tertiary Head and Neck Surgery Department over a 2-year period (2024-2025), with particular emphasis on cases of MPT. Data collected in **Table 2** included: sex, age, addictions, family history of cancer, relevant comorbidities, date of diagnosis of the first tumour, site of the index primary, histopathology, TNM, treatment (surgery/RT/CRT/systemic/combined), curative vs non-radical intent, date of diagnosis of the secondtumour, site, histopathology, TNM stage, treatment (surgery/RT/CRT/systemic/combined), curative vs non-radical intent, follow-up duration, and treatment outcomes.

**Table 2:** Clinical characteristics and outcomes of patients with multiple primary head and neck malignancies, who were treated in a tertiary head and neck surgery department (2024-2025).

Patient no.		Sex	Age	Addictions	FH	Comorbidities	DD	Site	HP	TNM	Treatment	Treatment intent	Follow up	Outcome
**I**		male	79	cigarettes	x	x							10 months	
	**T1**						mar.25	base of tongue	SCC	T2N1M0	CRT	curative		remission
	**T2**						apr.2025	liver	HCC	T2N0M0	ablation	non-radical		stable disease
**II**		male	72	cigarettes	x	x							12months	
	**T1**						nov.2024	larynx	SCC	T4N0M0	laryngectomy	curative		remission
	**T2**						dec.2024	liver	HCC	T3N0M0	ablation	non-radical		progression
**III**		male	70	cigarettes	x	HCV								
	**T1**						2008	parotid	pleomorph. Adenoma		surgery	non-radical	17y	2025 recurrence
	**T2**						aug. 2025	liver	HCC	T2N0M0	none - liver transplant after parotid surgery planned			
**IV**		male	69	cigarettes, alkohol	x	x								
	**T1**						mar.19	larynx	SCC	T4N1M0	laryngectomy+adj. RT	curative		
	**T2**						oct.2025	tonsil	SCC	T3N0M0	systemic	non-radical	3 months	progression
**V**		male	72	cigarettes, alkohol	x	x							ongoing	
	**T1**						jan.2025	larynx	SCC	T4N0M0	adj.RT + laryngectomy	curative		
	**T2**						dec.2025	base of tongue	SCC	T4N0M0	systemic	non- radical		
**VI**		male	62	cigarettes, alkohol	x	diabetes								
	**T1**						jan.2024	larynx	SCC	T1N0M0	TOLM	curative		remission
	**T2**						jan.2025	hypopharynx	SCC	T1N0M0	TORS	curative	12months	remission
**VII**		female	62	x	x	x								
	**T1**						oct.2022	larynx	SCC	T2N0M0	TOLM	curative		remission
	**T2**						feb.2025	parotid	Warthin	x	surgery	curative		remission
**VIII**		male	69	cigarettes, alkohol	x	obesity, diabetes							ongoing	
	**T1**						2014	larynx	SCC	T1N0M0	TOLM	curative		
	**T2**						2018	prostate	adenocarcinoma					
	**T3**						2023	kidney	oncocytoma					
	**T4**						oct.2025	larynx	SCC	T2N0M0	Rth	curative		tbd
**IX**		male	66	x	x	x								
	**T1**						oct.2019	larynx	SCC	T4N0M0	laryngectomy	curative		remission
	**T2**						feb.2025	parotid	Warthin	x	surgery	curative		remission
**X**		male	74	cigarettes, alkohol	x	diabetes, leg amputation, ABF								
	**T1**						aug. 2024	prostate	adenocarcinoma	T3bN0Mx	Rth, hormonotherapy			stable disease
	**T2**						feb. 2026	larynx	SCC	T4N3M+				
	**T3**						feb. 2026	lung	awaits					
**XI**		male	75			LVEF,								
	**T1**						feb. 2026	lung	awaits					
	**T2**						feb. 2026	pancreas	awaits					
	**T3**						feb. 2026	tonsil	SCC					

Bold values are: patient's number and tumor's number.

Multiple primary tumours (MPT) were defined according to the classical criteria of Warren and Gates, operationalised in line with contemporary IARC/SEER recommendations. For the purposes of this study, each lesion was required to meet the following conditions: (1) histological confirmation of malignancy; (2) a topographically and/or histologically distinct location, separated by clinically and radiologically normal mucosa from the index tumour where applicable; and (3) exclusion of the possibility that the additional lesion represented local recurrence, regional progression, or distant metastasis of the index cancer, based on integrated clinical, imaging, and histopathological assessment. Tumours fulfilling these criteria were classified as multiple primary tumours, irrespective of the time interval from the first diagnosis; timing (synchronous vs metachronous) was recorded and analysed separately.

### Data collection

Eligible patients were identified by a systematic search of the institutional electronic medical record and oncology registry for the predefined study period, using diagnostic codes for malignant head and neck tumours. Eligibility was confirmed by manual review of full medical records against the study inclusion and exclusion criteria.

Data were extracted with a standardised form, including demographics, primary tumour site and stage, histopathology, treatment, follow-up and the occurrence and characteristics of multiple primary tumours. Abstraction was performed by trained investigators and cross-checked by a second reviewer, with discrepancies resolved by consensus with a senior head and neck oncologist. Missing or inconsistent data were documented and, where critical, led to exclusion from specific analyses, as detailed in the Results. All data were anonymised before analysis in accordance with institutional and ethics requirements.

### Statistical analysis

Given the primarily descriptive and scenario-based nature of this work, statistical analyses were intentionally limited to basic descriptive summaries.

Drawing on published data and illustrative clinical examples, we present four scenarios:

1. Synchronous multiple primaries confined to the head and neck region;
2. Synchronous head and neck cancer with a distant primary;
3. Metachronous primaries, both within the head and neck;
4. Metachronous head and neck cancer combined with a non-head and neck malignancy.

For each scenario, we present clinical cases and outline diagnostic priorities, multidisciplinary decision-making and contemporary treatment strategies in the era of IMRT, re-irradiation and immunotherapy.

## Results

Over the study period, 274 patients with HNSCC were treated, of whom 15 had MPT. The characteristics of patients with MPT are summarised in **Table 2**. Among 274 consecutively treated patients with HNSCC, 15 individuals met criteria for multiple primary tumours, corresponding to approximately 5-6% of the cohort.

**Table 2** details the clinical courses of patients who developed more than one malignancy, thereby exemplifying both metachronous and, in selected instances, synchronous constellations of HNC within the upper aerodigestive tract. The cohort is dominated by older male smokers, frequently with concomitant alcohol misuse and relevant comorbidities such as diabetes, obesity or chronic viral hepatitis, which is consistent with the high risk phenotype for second primary tumours described in large head and neck series. First primaries were most often treated with curative intent surgery, including transoral laser microsurgery or open laryngectomy with or without adjuvant radiotherapy, resulting in durable local control at the time the subsequent HNC was diagnosed. In contrast, the second HNCs in this table span a wide TNM spectrum- from early T1N0 lesions amenable to minimally invasive salvage (TOLM/TORS) to advanced T3–T4 carcinomas requiring extensive surgery or nonradical systemic therapy- illustrating how prior treatment and cumulative functional impairment may constrain the feasibility of fully curative strategies. Notably, patients presenting with a second HNC at an early stage and managed surgically tended to maintain remission in short to intermediate term follow up, whereas those with bulky or node positive disease were more likely to experience early progression despite aggressive. multimodality treatment, in line with published data on the poor prognosis of advanced second primaries in this population.

**Table 3** illustrates patients harbouring combinations of HNC with extracephalic malignancies, encompassing both synchronous and metachronous multiple primaries involving the liver, prostate, kidney, lung, and pancreas. This subset underscores a particularly complex clinical scenario in which the index HNSCC frequently achieves locoregional remission after standard curative intent treatment, whilst subsequent visceral tumour- most notably hepatocellular carcinoma and advanced prostate cancer- often dictate overall prognosis by virtue of their stage and limited therapeutic window. Several patients exemplify a cascading pattern of malignancy, with sequential primaries in different organs over many years, reflecting the combined impact of field cancerisation, persistent carcinogenic exposure and age-related comorbidity, as described in contemporary series of multiple primary malignancies. Treatment strategies in these cases range from organ-confined, site-specific radical approaches (e.g. ablative therapy for early HCC or definitive radiotherapy with hormonal manipulation for prostate cancer) to clearly nonradical or purely palliative regimens in the setting of disseminated disease, highlighting how the therapeutic intent must be continuously realigned with the tumour that currently dominates survival and symptom burden. Collectively, the trajectories depicted in **Table 3** make apparent that, in patients with HNSCC and additional head and neck primaries, the feasibility of combined curative management is often restricted, and decision-making must integrate organ specific guidelines with a realistic appraisal of cumulative toxicity and global functional reserve.

**Table 3:** Clinical matrix of multiple primary malignancies involving head and neck cancer/.

	Synchronous primaries	Metachronous primaries
**Both tumours in head and neck**	**Synchronous HNC + HNC**	**Metachronous HNC + HNC**
	Diagnostics: Panendoscopy, comprehensive head and neck imaging, histology ± HPV/p16, bilateral nodal staging.	Diagnostics: Differentiate recurrence from new primary; review prior RT fields/doses and surgical reports; endoscopy of scarred/irradiated mucosa; new imaging with comparison to baseline.
	Multidisciplinary decision-making: Head and neck tumour board to design a single combined curative strategy; early discussion of airway security and reconstruction.	Multidisciplinary decision-making: Tumour board focus on feasibility of salvage surgery vs re-irradiation; integrate geriatric and functional assessment.
	Treatment sequencing: Prefer a unified surgical and/or RT plan covering both lesions; stage procedures only if needed for airway or complex reconstruction.	Treatment sequencing: Sequential by definition; second tumour treated with full awareness of prior toxicity and organ reserve.
	Prognosis: Dominated by the more advanced/aggressive tumour; high risk of functional impairment (swallowing, speech) despite curative intent.	Prognosis: Better with long disease-free interval and early-stage second tumour; worse with short interval, heavy prior treatment and poor baseline function.
**One HNC, one extra-HNC tumour**	**Synchronous HNC + extra-HNC**	**Metachronous HNC + extra-HNC**
	Diagnostics: Parallel staging of both sites with site-specific imaging; histology/IHC to rule out metastasis; assessment of performance status and comorbidities.	Diagnostics: Confirm second primary (not metastasis) by histology and imaging pattern; stage the new cancer; evaluate cumulative treatment burden and current organ function.
	Multidisciplinary decision-making: Joint head and neck plus organ-specific board (e.g. hepatobiliary, urology, breast); define an overarching treatment goal and acceptable toxicity.	Multidisciplinary decision-making: Decide which malignancy currently drives prognosis and symptoms; align site-specific guidelines with global fitness and life expectancy; involve palliative care early in advanced cases.
	Treatment sequencing: Treat first the tumour with greatest short-term risk (airway obstruction, bleeding vs visceral failure); consider near-simultaneous therapy if organ-at-risk constraints allow.	Treatment sequencing: Apply standard site-specific treatment for the second tumour if early stage and the first is controlled; if one cancer is recurrent or advanced, prioritize the disease with higher impact on survival and symptom burden.
	Prognosis: Determined by the interaction of two independent diseases; dual advanced stage often limits curative options and pushes management towards palliation.	Prognosis: Favourable when both primaries are detected early and treated definitively; advanced stage of either tumour, short interval and major comorbidity reduce overall survival and shift focus to symptom control.

Bold values are: patient's number and tumor's number.

## Discussion

Multiple primary malignancies involving the head and neck epitomise the interplay of field cancerisation, treatment-induced tissue vulnerability and the demographic shift towards an older, multimorbid cancer population (7–10, 22–28, 31, 32). In the modern era, IMRT, sophisticated re- irradiation protocols and immune checkpoint blockade have expanded the range of local and systemic strategies available for these patients, but they have also made decision-making more complex and highly individualised (22–32).

This work emphasises the need to move from anecdotal case reporting towards a structured, scenario-based management paradigm for multiple primary tumours involving the head and neck. By demonstrating that 15 of 274 consecutively treated HNSCC patients met criteria for MPT over just two years, it highlights that this is not a rare curiosity but a recurrent workload for tertiary head and neck units, demanding formalised pathways rather than *ad hoc* decisions.

### Scenario 1: synchronous primaries confined to the head and neck

#### Diagnostic priorities

Synchronous primaries limited to the head and neck, such as a laryngeal cancer with a simultaneous oral cavity or oropharyngeal tumour, are prototypical manifestations of field cancerisation (1, 2, 6–10). Thorough diagnostic work-up is essential to avoid understaging. It should include:

- Panendoscopy with systematic inspection and biopsy of all suspicious sites (1, 2, 6);
- Contrast-enhanced CT and/or MRI of the entire upper aerodigestive tract, with PET-CT when indicated (2, 5, 6);
- Histopathologic confirmation of each lesion, including HPV/p16 assessment for oropharyngeal tumours (33–37);
- Comprehensive nodal staging with bilateral neck imaging and ultrasound-guided FNA as needed (2, 5, 6).

Such a work-up helps distinguish between two separate primaries and multifocal growth within a single tumour field, which has implications for prognosis and treatment planning (8, 19–21).

#### Multidisciplinary decision-making

The central multidisciplinary questions in this scenario are whether a single curative-intent plan (surgical and/or IMRT-based) can adequately address both primaries and the neck, which lesion poses the greater immediate threat (airway obstruction, bleeding, aspiration), and what functional trade-offs, particularly for speech and swallowing, are acceptable to the patient. IMRT enables simultaneous treatment of multiple mucosal targets and nodal basins with improved sparing of salivary glands, pharyngeal constrictors and spinal cord compared with older approaches (22–28). This allows integrated plans that encompass, for example, laryngeal and oropharyngeal primaries within a single course of chemoradiotherapy, potentially avoiding sequential courses of RT, whilst surgery may be considered for selected accessible lesions (2, 5, 6, 22–28).

#### Treatment strategies in the IMRT era

In fit patients without distant metastases, curative-intent treatment is usually appropriate. Typical strategies include:

- Combined resection of both primaries with appropriate neck dissection(s), followed by risk-adapted adjuvant IMRT with or without systemic therapy;
- Surgery for one lesion and definitive IMRT-based chemoradiotherapy for the other (2, 5, 6, 38);
- Definitive IMRT or chemoradiotherapy to both sites using a carefully individualised target volume design (22–28, 38).

Immune checkpoint inhibitors have transformed systemic therapy in recurrent/metastatic HNSCC, but their upfront role in resectable synchronous primaries remains investigational (29, 30). In patients who present with synchronous primaries and incurable metastatic disease, pembrolizumab or nivolumab-based regimens may take precedence over extensive locoregional treatment (29, 30).

### Scenario 2: synchronous head and neck cancer and a distant primary

#### Diagnostic priorities

Synchronous HNSCC and a distant primary, such as intrahepatic cholangiocarcinoma, prostate or breast cancer, pose distinct diagnostic and therapeutic challenges. The first priority is to determine whether the distant lesion represents a metastasis or an independent primary (3–5, 19–21), using:

- Organ-specific imaging and staging where appropriate, for example liver protocol CT/MRI in suspected biliary tract malignancy (39, 40);
- Histologic and immunohistochemical comparison (4, 5);
- Assessment of performance status and comorbidities to estimate tolerance for combined therapy (31, 32).

#### Multidisciplinary decision-making and sequencing

Optimal management requires close coordination between the head and neck oncology team and the relevant organ-specific tumour board. Key decisions include which tumour poses the greatest short-term risk (airway compromise, hepatic failure, spinal cord compression), whether curative options are realistically available for one or both tumours, and whether treatments can be delivered concurrently without unacceptable cumulative toxicity (4, 5). For example, definitive treatment of HPV-positive OPC may need to be sequenced with liver-directed treatment for early-stage intrahepatic cholangiocarcinoma (33–37, 39, 40), whereas some indolent second primaries may reasonably be deferred whilst priority is given to curative treatment of the head and neck malignancy (4, 5).

#### Role of systemic therapy, targeted agents and immunotherapy

The presence of synchronous primaries complicates the selection of systemic therapy. Head and neck disease may warrant platinum-based chemotherapy and/or pembrolizumab (29, 30, 38), whilst concomitant malignancies may require endocrine therapy, HER2-targeted agents or other organ- specific systemic treatments. Careful coordination is required to avoid overlapping toxicities and to manage potential drug–drug interactions, particularly when combining checkpoint inhibitors for more than one indication (4, 5).

### Scenario 3: metachronous primaries within the head and neck

#### Diagnostic and classification issues

Metachronous head and neck primaries, such as a new laryngeal or oropharyngeal carcinoma arising years after treatment of an initial HNSCC, are difficult to distinguish from late local recurrences (1, 4–6, 19–21, 28). Determining whether a new lesion constitutes a second primary or a recurrence requires:

- Review of previous operative reports, radiotherapy fields and cumulative doses (22–28);
- Endoscopic assessment of the entire upper aerodigestive tract including scarred and irradiated mucosa (1, 2, 6);
- New contrast-enhanced imaging with direct comparison to baseline studies (2, 5, 6);
- Biopsy with detailed histologic evaluation, with HPV status or other biomarkers in selected cases to support the diagnosis of an independent primary (33–37).

From a practical perspective, however, the distinction mainly affects coding and prognosis; the key therapeutic question is whether a second curative-intent approach is feasible given prior treatment (3, 19–21).

#### IMRT-based re-irradiation with or without immunotherapy

Historically, re-irradiation in previously treated head and neck fields was associated with modest survival and high rates of severe toxicity, including carotid blowout and soft-tissue necrosis. The introduction of IMRT has improved target coverage and normal-tissue sparing, enabling more precise re-irradiation in selected patients (22–28). In parallel, nivolumab and pembrolizumab have broadened systemic salvage options in recurrent/metastatic HNSCC (29, 30). The combination of re-irradiation and immunotherapy remains an area of active clinical exploration, but robust long- term data are still limited. Candidate selection remains critical, and prior dose distributions, cumulative doses to the spinal cord, brainstem, carotids, mandible and pharyngeal musculature, as well as vascular anatomy, must be scrutinised (25–28); for many patients, particularly those with extensive prior RT, severe late toxicity or poor performance status, re-irradiation will not be appropriate, and systemic therapy or palliative RT may represent the only realistic options (22– 28).

#### Salvage surgery and perioperative systemic therapy

Where anatomically and functionally feasible, salvage surgery remains the cornerstone of curative management for metachronous primaries in previously irradiated fields (2, 5, 6, 22–28, 41). Total laryngectomy, partial pharyngolaryngectomy or circumferential pharyngectomy may achieve long-term control in carefully selected patients, albeit at the cost of substantial functional impairment and morbidity (41). The role of perioperative systemic therapy, including immunotherapy, in this setting is evolving: early-phase trials are exploring neoadjuvant and adjuvant checkpoint inhibition in high-risk HNSCC to reduce microscopic residual disease and distant metastases, but robust data specific to metachronous primaries or re-irradiation contexts are not yet available (29, 30).

### Scenario 4: metachronous head and neck cancer with a non-head and neck malignancy

#### Diagnostic, geriatric and survivorship issues

Many patients with metachronous HNSCC are older cancer survivors with a history of prior malignancies, such as prostate, breast or colorectal cancer, treated years earlier (4, 5, 31, 32). In this setting, the feasibility and desirability of aggressive HNSCC treatment depend on the status and prognosis of the prior cancer (cured vs active vs indolent), the cumulative treatment burden and a careful evaluation of performance status, comorbidities and geriatric parameters (4, 5, 31, 32). Comprehensive geriatric assessment (CGA) has been shown to improve treatment selection and survival in older cancer patients and is increasingly recommended for elderly HNSCC patients when major treatment decisions are contemplated (31, 32).

#### Role of immunotherapy and targeted agents

For patients with recurrent/metastatic HNSCC arising after treatment for another primary cancer, immunotherapy has become a key systemic option. Pembrolizumab, alone or in combination with platinum/5-FU, is the first-line standard based on KEYNOTE-048 (30), whilst nivolumab has shown benefit in the post-platinum setting (29). For older, multimorbid patients, these regimens may offer a more favourable risk–benefit ratio than additional lines of cytotoxic chemotherapy, particularly when the therapeutic goal is durable disease control with preservation of quality of life (29–32). At the same time, clinicians must account for ongoing or prior targeted therapies or endocrine regimens for the first malignancy. Current epidemiologic data do not support a strong association between androgen deprivation therapy and HNSCC, although ADT may modify the risk profile for some second malignancies (42).

### Practical framework

To translate these concepts into clinical practice, we propose viewing multiple primary head and neck cancers through a 2 × 2 clinical matrix (synchronous vs metachronous; both HNC vs HNC plus extra-HNC), complemented by scenario-specific checklists covering:

- Diagnostic work-up: endoscopy, imaging, pathology, HPV/p16;
- Multidisciplinary decision-making: which tumour drives prognosis, feasibility of curative-intent treatment, and geriatric assessment;
- Local treatment options: surgery, IMRT, re-irradiation;
- Systemic therapy choices: cisplatin, immunotherapy, targeted agents;
- Prognostic/quality-of-life considerations (1–6, 19–32, 38).

Such a structured approach can support tumour board deliberations and help align complex therapeutic decisions with patient values and realistic expectations (1–6, 31, 32).

Organisationally, the article argues that MPT care should be anchored in systematic diagnostic algorithms (including panendoscopy, high-quality cross-sectional imaging and meticulous histopathology), embedded within tumour board-driven decision-making that explicitly weighs curative potential, toxicity of retreatment and functional outcomes across four recurring clinical scenarios (synchronous vs metachronous, intra- vs extra-cephalic) (1–6, 22–32). It also illustrates that contemporary technologies — IMRT, re-irradiation protocols and checkpoint inhibition — expand curative and salvage options but require coordinated sequencing between head and neck teams and other organ-specific boards when distant primaries coexist (22–30, 39, 40). Clinically, the proposed 2 × 2 matrix with scenario-specific checklists provides a pragmatic framework for standardising work-up, clarifying which tumour drives prognosis and guiding the choice between surgery, IMRT (including re-irradiation), systemic therapy and palliative approaches in older, often multimorbid survivors (1–6, 22–32, 38). In doing so, the paper positions multiple primary head and neck cancers as a distinct, complex care domain that warrants dedicated protocols, longitudinal survivorship planning and explicit integration of geriatric assessment into routine multidisciplinary practice (31, 32).

To summarize, in patients with synchronous malignancies, therapeutic priorities should follow a hierarchy shaped by three axes: (a) imminent threats to life or organ function (airway compromise, catastrophic bleeding, impending hepatic or respiratory failure), (b) the feasibility of delivering a single, truly curative strategy that encompasses both tumours, and the magnitude of anticipated functional loss, explicitly weighed against the patient’s values and preferences (2, 5, 6, 22–32). In practice, the lesion that drives short-term mortality or irreversible morbidity becomes the primary determinant of sequencing, but whenever organ reserve and cumulative toxicity thresholds permit, parallel or tightly sequenced curative-intent treatment of both cancers should be pursued rather than accepting an *a priori* palliative trajectory (2, 5, 6, 22–32). The scenario-based 2 × 2 matrix derived from this cohort operationalises this hierarchy by forcing the multidisciplinary team to identify, for each case, which tumour governs prognosis and symptom burden and whether a unified curative plan is realistic, or whether care must pivot to staged or purely palliative strategies (1–6). Embedding this matrix and its scenario-specific checklists into routine tumour board deliberations would transform the management of synchronous primaries from *ad hoc* improvisation into a reproducible, transparent decision pathway when head and neck teams must coordinate with other organ-specific boards (1–6, 39, 40).

Regarding surveillance, both the literature and the present series indicate that the traditional 5-year follow-up horizon is poorly aligned with the biology of field cancerisation, as a substantial proportion of metachronous, unresectable or non-radically treatable primaries emerge beyond the fifth year (7, 9, 10, 43, 44). Long-term studies in HNSCC populations demonstrate a sustained risk of second primaries over many years, which argues for extending structured specialist surveillance, particularly in current or former smokers and in patients previously exposed to high-dose radiotherapy (33, 43, 44). In this context, a two-phase survivorship model appears more rational than a hard 5-year cut-off: an initial intensive phase focused on detection of recurrence and early metachronous disease, followed by a prolonged, lower-intensity phase explicitly aimed at second primaries, late toxicities and risk-factor modification (43, 44). For high-risk survivors of HNSCC, the data and the current cohort together support framing follow-up not as “5-year cure” but as long- term, potentially lifelong oncologic stewardship, with visit frequency and diagnostic intensity modulated by age, comorbidity, exposure history and prior treatment burden (43, 44).

## Conclusions

- Multiple primary head and neck tumours are a clinically significant problem, affecting ~5- 6% of HNSCC patients in a contemporary tertiary cohort.
- Management should shift from case-by-case improvisation to a scenario-based framework (2 × 2 matrix: synchronous vs metachronous; intra- vs extra-head-and-neck) with standardised diagnostic and decision-making checklists.
- Modern modalities- IMRT (including re-irradiation) and immune checkpoint inhibitors expand curative and salvage options but demand tightly coordinated, tumour board-driven sequencing with organ-specific teams.
- Given the predominance of older, multimorbid survivors, systematic incorporation of comprehensive geriatric assessment is essential to align treatment intensity with prognosis, toxicity risk, and patient-reported goals.
- The observed temporal patterns of non-resectable metachronous primaries arising beyond the conventional 5-year surveillance window argue against a rigid 5-year boundary and support reconceptualising follow-up for HNSCC survivors as a long-term, risk-adapted survivorship pathway explicitly targeting second primaries.
- This work proposes a structured, scenario-based framework for approaching patients with multiple primary tumours in head and neck oncology, integrating current evidence with illustrative data from a single-centre institutional cohort. The framework is intended as a;
- hypothesis-generating and educational tool to support clinical reasoning, rather than as a definitive or prescriptive algorithm, and should be adapted cautiously to local practice. Its assumptions and clinical utility require prospective and preferably multicentre validation in larger populations before any firm recommendations can be made.

## Limitations

This study has several limitations that should be considered when interpreting its findings. It is a retrospective analysis from a single tertiary centre with a relatively small number of patients with multiple primary tumours, which limits statistical power and generalisability. Despite applying established criteria and multidisciplinary review, some misclassification between multiple primaries, recurrences and metastases cannot be entirely excluded. Moreover, the institutional cohort was used mainly to illustrate a scenario-based framework rather than to provide definitive comparative or prognostic evidence. Consequently, the proposed framework should be regarded as hypothesis-generating and requires validation in larger, preferably multicentre prospective studies before firm clinical recommendations can be made.

## Data Availability

The original contributions presented in the study are included in the article/supplementary material. Further inquiries can be directed to the corresponding author.
